# The interaction of celecoxib with MDR transporters enhances the activity of mitomycin C in a bladder cancer cell line

**DOI:** 10.1186/1476-4598-12-47

**Published:** 2013-05-24

**Authors:** Vincenzo Pagliarulo, Patrizia Ancona, Mauro Niso, Nicola Antonio Colabufo, Marialessandra Contino, Luigi Cormio, Amalia Azzariti, Arcangelo Pagliarulo

**Affiliations:** 1 Sezione di Urologia e Andrologia, Dipartimento dell’Emergenza e dei Trapianti di Organi (DETO), Università “Aldo Moro” di Bari, Piazza G. Cesare 11, Bari 70124, Italy; 2 Dipartimento Farmacochimico, Università “Aldo Moro” di Bari, Via Orabona, 4, Bari 70125, Italy; 3 Sezione di Urologia e Trapianti di Reni, Università di Foggia, Viale Pinto 1, Foggia 71100, Italy; 4 Istituto Tumori “Giovanni Paolo II” IRCCS Ospedale Oncologico, Viale Orazio Flacco 65, Bari 70124, Italy

**Keywords:** Cyclooxygenase-2, Multidrug resistance, Bladder cancer

## Abstract

**Background:**

An *in vitro* model was developed to understand if celecoxib could synergize with Mitomycin C (MMC), commonly used for the prevention of non-muscle invasive bladder cancer recurrence, and eventually elucidate if the mechanism of interaction involves multi drug resistance (MDR) transporters.

**Methods:**

UMUC-3, a non COX-2 expressing bladder cancer cell line, and UMUC-3-CX, a COX-2 overexpressing transfectant, as well as 5637, a COX-2 overexpressing cell line, and 5637si-CX, a non COX-2 expressing silenced 5637 cell line, were used in the present study. The expression of COX-2 and MDR pumps (P-gp, MDR-1 and BCRP) was explored through western blot. The anti-proliferative effect of celecoxib and MMC was studied with MTT test. Three biological permeability assays (Drug Transport Experiment, Substrate Transporter Inhibition, and ATP cell depletion) were combined to study the interaction between MDR transporters and celecoxib. Finally, the ability of celecoxib to restore MMC cell accumulation was investigated.

**Results:**

The anti-proliferative effect of celecoxib and MMC were investigated alone and in co-administration, in UMUC-3, UMUC-3-CX, 5637 and 5637si-CX cells. When administered alone, the effect of MMC was 8-fold greater in UMUC-3. However, co-administration of 1 *μ*M, 5 *μ*M, and 10 *μ*M celecoxib and MMC caused a 2,3-fold cytotoxicity increase in UMUC-3-CX cell only. MMC cytotoxicity was not affected by celecoxib co-administration either in 5637, or in 5637si-CX cells. As a result of all finding from the permeability experiments, celecoxib was classified as P-gp unambiguous substrate: celecoxib is transported by MDR pumps and interferes with the efflux of MMC. Importantly, among all transporters, BCRP was only overexpressed in UMUC-3-CX cells, but not in 5637 and 5637si-CX.

**Conclusions:**

The UMUC-3-CX cell line resembles a more aggressive phenotype with a lower response to MMC compared to the *wt* counterpart. However, the administration of celecoxib in combination to MMC causes a significant and dose dependent gain of the anti-proliferative activity. This finding may be the result of a direct interaction between celecoxib and MDR transporters. Indeed, BCRP is overexpressed in UMUC-3-CX, but not in UMUC-3, 5637, and 5637si-CX, in which celecoxib is ineffective.

## Background

The natural history of non-muscle invasive bladder carcinoma (NMIBC) may be characterized by multiple recurrences according to patient risk stratification [1]. Following transurethral resection (TUR), endovesical chemotherapy is recommended by clinical practice guidelines in intermediate and high-risk patients [2]. Mitomycin C (MMC) is a potent DNA cross-linker commonly used as a bladder instillation to reduce the likelihood of bladder cancer recurrence and/or progression [3]. Nonetheless, early recurrence is common after MMC treatment and may recognize several causes, such as disease phenotype [1], the presence of residual tumor following a TUR [4], as well as downstaging [5]. Another possible cause may be the failure of cancer treatment due to resistance to structurally unrelated chemotherapeutic agents, also defined Multi Drug Resistance (MDR) [6,7]. MDR is usually associated with a decreased intracellular concentration of cytostatic drugs and with the overexpression of ATP-Binding Cassette (ABC) transporters, such as P-glycoprotein (P-gp), Breast Cancer Resistance Proteins (BCRP) and Mutidrug Resistance associated Proteins (MRPs) [8-10]. These transporters cause the efflux of drugs and xenobiotics out from the cells [11]. Interestingly, their overexpression has been observed in urothelial cancer cells [12] and may be useful for selecting patients with bladder cancer to be candidates for neoadjuvant chemotherapy [13].

Cyclooxygenases (COX) are enzymes required for the conversion of arachidonic acid to prostaglandins. As COX-1 is constitutively expressed, COX-2 is highly induced in response to inflammatory signals [14]. Although specific COX-2 inhibitors were generated for pain relief and for their anti-inflammatory properties, experimental and translational studies have shown that COX-2 is involved in carcinogenesis, finally encouraging clinical testing [15]. Recently, COX-2 inhibitors have gained attention as chemosensitizers when combined with other agents [16,17]. This property may be exerted by interference with the activity of membrane proteins involved in MDR [18,19].

In the urinary bladder, COX-2 plays an important role in the development of transitional cell hyperplasia and carcinoma [20]; COX-2 expression in the urothelium is associated with high tumor grade and stage, and is an independent predictor of disease progression and survival [21,22]. Although treating these patients with endovesical MMC causes a reduction in the recurrence and progression rates of NMIBC, disease relapse is still high [2]. Since COX-2 inhibitors, such as celecoxib, may sensitize cells to antineoplastic agents we sought to investigate if there is an interaction between celecoxib, MMC and MDR transporters in a human bladder cancer cell line.

## Methods

### Cell lines and cell culture

UMUC-3, 5637, TCC sup cells and J82 (ATCC, Manassas, VA, USA), derived from human transitional cell carcinoma of the bladder, were routinely cultured in MEM supplemented with 10% fetal bovine serum, 2 mM glutamine, 100 U/mL penicillin, 100 μg/mL streptomycin, 1X NEAA and 1 mM sodium pyruvate. All components were purchased form Invitrogen Corportion (Cergy Pontoise, France), in a humidified incubator at 37°C with a 5% CO2 atmosphere. Caco-2 (IRCCS “S. De Bellis”, Castellana Grotte, Italy), MDCK-MDR1, MDCK-MRP1 (gift of Prof. P. Borst, NKI-AVL Institute, Amsterdam, Netherlands) and MDCK-BCRP cells (gift of Dr. A. Schinkel, NKI-AVL Institute, Amsterdam, Netherlands) were grown in DMEM medium with 10% heat-inactivated fetal calf serum, 100 U/mL penicillin, 100 μg/mL streptomycin and 2 mM L-glutamine (Invitrogen) in a humidified incubator at 37°C with a 5% CO2 atmosphere. The cells were trypsinized twice a week with trypsin/ethylenediaminetetraacetic acid (EDTA) (0.05%/0.02%) and the medium was changed twice a week.

### Cell lines transfection

The pSG5-COX-2 plasmid, which contains a full-length human COX-2 cDNA in the pSG expression vector [23], was used for COX-2 transfection. The plasmid DNA was introduced into UMUC-3 cells using Lipofectamine LTX (Invitrogen). Briefly, 2.5 μg of plasmid DNA and 6.25 μl of Lipofectamine LTX were combined in 500 μl of OpTI-MEM*I reduced serum medium (Invitrogen) and allowed to stand at room temperature for 30 minutes. UMUC-3 cells were plated at 1X105 cells/well in six-well plates. After overnight attachment, the Lipofectamine mixture was applied to cells. After 5 hours of transfection, the medium was removed and complete medium was added. After 48 hours, geneticin G418 (Invitrogen) was added to cells at a concentration of 800 mg/ml. Cell lines were obtained from individual colonies using cloning cylinders. UMUC-3-CX overexpressing cells were obtained and were continuously cultured in complete medium with the addition of 800 μg/ml G418.

### Cell lines siRNA transfection

The COX-2 si RNA, target- specific 19–25 nt siRNA designed to knock down gene expression, was used for the inihibition of COX-2 expression in 5637 bladder cells. The COX-2 siRNA (Santa Cruz Biotech., CA, USA) was introduced into 5637 cells using siRNA Transfection Reagent (Santa Cruz Biotech.) Briefly, 5 μl of siRNA and 5 μl of siRNA Transfection Reagent were combined in 1 ml of siRNA Transfection Medium (Santa Cruz) and allowed to stand at room temperature for 30 minutes. 5637 cells were plated at 2X10^5^ cells/well in six-well plates. After overnight attachment, the siRNA Transfection Reagent mixture was applied to cells. After 5 hours of transfection, the medium was removed and complete medium was added.

### Prostaglandin E_2_ (PGE2) quantification

PGE2 concentration was determined in the culture medium of UMUC-3, UMUC-3-CX, 5637, 5637si-CX, TCC sup and J82, cells. The cells were seeded into 24-well plates in the absence and presence of celecoxib (1- 5–50 μM) into a final volume of 500 μl/well of standard growth medium and incubated at 37°C for 24–48 h. After incubation time, the culture media were removed to determine PGE2 levels with the PGE2 monoclonal enzyme immunoassay kit (Cayman Chemicals) according to the manufacturer’s protocol and results are expressed in pg of PGE2/ml of medium.

### Western immunoblotting

UMUC-3, UMUC-3-CX, 5637, 5637si-CX, TCC sup, and J82 cells were washed with 10 mL phosphate-buffered saline (PBS), scraped in 1 mL PBS and centrifuged for 1 min at 11,000g. Proteins were extracted from cells by homogenization in radioimmunoprecipitation (RIPA) buffer [0.5 M NaCl, 1% Triton X100, 0.5% NP40, 1% deoxycolic acid, 3.5 mM sodium dodecyl sulfate (SDS), 8.3 mM Tris HCl pH 7.4, 1.6 mM Tris base] and treated with 20% protease inhibitor cocktail (Sigma Aldrich, St. Louis, MO, USA). They were sonicated and centrifuged at 14,000 g for 15 min at 4°C and the protein content in the supernatant was measured using the Bradford method. 25–50 μg of protein extract was separated electrophoretically on 8% SDS–polyacrylamide gel electrophoresis (SDS–PAGE) and proteins electroblotted onto a nitrocellulose membrane (Bio-Rad, Hercules, CA, USA). Membranes were stained with 0.5% ponceau in 1% acetic acid to confirm equal loading. After overnight incubation of the membranes in blocking buffer (5% non-fat dried milk, 0.1% Tween 20 in Tris-buffered salt solution, TBS) they were incubated overnight with the respective primary antibody directed against COX-2 (1:500 mouse monoclonal), P-gp (1:1000 mouse monoclonal), MRP1 (1:1000 mouse monoclonal), BCRP (1:1000 mouse monoclonal) diluted in blocking buffer. The COX-2 antibody was purchased from Cayman Chemical (Ann Arbor, MI, USA), all other antibodies from Sigma-Aldrich. Membranes were washed four times with 0.1% Tween 20 in TBS and then incubated with a peroxidase-conjugated secondary antibody (Amersham, Buckinghamshire, UK) for1 hr. After extensive rinsing in 0.1% Tween 20 in TBS, protein–antibody complexes conjugated with peroxidise were treated with enhanced chemoluminiscence (ECL-Plus, Amersham) according to the manufacturer’s protocol and exposed to a chemoluminescence film. The expression level was evaluated by densitometric analysis using Quantity One software (Bio-Rad) and β-actin expression level was used to normalize the sample values.

### Flow cytometry

UMUC-3 and UMUC-3-CX cells were harvested, washed twice in ice-cold PBS (pH 7.4), fixed in 4.5 mL of 70% ethanol, and stored at −20°C. Fixed cells were washed in ice-cold PBS once and incubated in 0.5 mL of 0.1% Tween 20 in PBS for 15 minutes at 25°C. To analyze P-gp/MRP1/BCRP expression, UMUC-3 and UMUC-3-CX cells were incubated O/N at 4°C with monoclonal anti-BCRP in 0.5% Tween 20 and 1% FBS in PBS. To determine the non-specific fluorescence due to the fluorescein conjugated secondary antibody, UMUC-3 and UMUC-3-CX cells were incubated with an appropriate isotype control (50 Ag/106 cells) in the same experimental conditions (isotype control). After 15 minute incubation with 0.5 mL of 0.5% FBS in PBS, cells were centrifuged and washed once in 0.5 mL of 0.5% FBS in PBS. The pellet was resuspended in 0.5% FBS in PBS in the presence of the goat anti-mouse IgG fluorescein-conjugated affinity-purified secondary antibody (Millipore, Billerica, MA, USA; 1:50) and incubated for 1 hour at 4°C. After a wash step with 0.5 mL of 0.5% FBS in PBS, cells were centrifuged and incubated in 5 Ag/mL propidium iodide overnight at 4°C. BCRP protein determination was done using a FACScan flow cytometer (Becton Dickinson, Franklin Lakes, NJ, USA). Fluorescence analysis was gated to include single cells based on forward and side light scatter and was based on the acquisition of data from 10,000 cells. Log fluorescence was collected and displayed as single variable histograms. The data analysis was carried out with the CellQuest software (Becton Dickinson).

### Cell anti-proliferative effect

The cells were seeded into 96-well plates in the absence and presence of known concentrations (0,1-0,5-1-5-10-30-50 μM) of celecoxib (Pfizer, NY, USA) or MMC (Kyowa, Dusseldorf, DE), alone and in co-administration, added to a final volume of 200 μl/well of standard growth medium and incubated at 37°C for 48 h. Afterwords, 10 μl of 3-[4,5-dimethylthiazol-2-yl]-2,5-diphenyltetrazoliumbromide (MTT) freshly prepared solution (5 mg/ml in PBS) (Sigma) was added in each well, and the plate was incubated in a humidified atmosphere 5% CO2 at 37° for 3–4 h. MTT solution was removed and 200 μL of EtOH/DMSO (1:1) was added to each well to dissolve the blue formazan solid crystals. The optical density was measured at 570 nm and 650 nm wavelenghts using Victor3 (PerkinElmer, Waltham, MA, USA). The results are expressed as EC50 values, obtained from non-linear iterative curve fitting by Prism v.3.0, GraphPad software (GraphPad Software, Inc. San Diego, USA).

### Permeability experiments

In the following three experiments a Caco-2 cell monolayer was used to evaluate P-gp transporter’s interaction with a given compound. In the “drug transport experiment” the ability of a compound to permeate a cellular monolayer is tested. Both apical (AP)→basolateral (BL) flux and BL→A flux are evaluated since their ratio determines the permeability value: compounds with a permeability ratio > 2 do not permeate, compared to compounds with a ratio < 2. In the “[3H]Substrate transport inhibition” assay, a given compound competes for P-gp with its known substrate, [3H]vinblastine. If our compound (i.e. celecoxib) binds P-gp, [3H]vinblastine will be displaced reducing residual radioactivity. Finally, since P-gp is an ATPasic pump, the “Cell ATP availability assay” will test if cells consume ATP in the presence of a given compound. P-gp binding compounds are transported out of the cell and produce ATP consumption. Taken together, these assays allow us to define if a compound is either a transporter’s substrate, inhibitor, or modulator, according to Polli’s classification [24].

### Preparation of caco-2 monolayer

Caco-2 cells were seeded onto a Millicell® assay system (Millipore), where a cell monolayer is set in between a filter cell and a receiver plate, at a density of 10,000 cells/well. The culture medium was replaced every 48 h and the cells kept for 21 days in culture. The Trans Epitelial Electrical Resistance (TEER) of the monolayers was measured daily, before and after the experiment, using an epithelial voltohometer (Millicell® -ERS). Generally, TEER values greater than 1000 Ω for a 21day culture, are considered optimal.

### Drug transport experiment

After 21 days of Caco-2 cell growth, the medium was removed from filter wells and from the receiver plate, which were filled with fresh HBSS buffer (Invitrogen). This procedure was repeated twice, and the plates were incubated at 37°C for 30 min. After incubation time, the HBSS buffer was removed and drug solutions of celecoxib, MMC and reference compounds, were added to the filter well at various concentrations (1–100 μM), while fresh HBSS was added to the receiver plate. The plates were incubated at 37°C for 120 min. Afterwords, samples were removed from the apical (filter well) and basolateral (receiver plate) side of the monolayer to measure the permeability [24]. The apparent permeability (Papp), in units of nm/second, was calculated using the following equation:

$$p\;=\left[\frac{V_{A}}{\mathrm{Area}\;\times \;\mathrm{time}}\right]\;\times \;\left[\frac{\left[\mathrm{drug}\right]\mathrm{acceptor}}{\left[\mathrm{drug}\right]\mathrm{initial}}\right]$$

VA = the volume (in mL) in the acceptor well;

Area = the surface area of the membrane (0.11 cm2 of the well);

time = the total transport time in seconds (7200 sec);

[drug]acceptor = the concentration of the drug measured by ESI-MS analyses or U.V. spectroscopy; [drug]initial = the initial drug concentration (1 × 10–4 M) in the apical or basolateral wells.

### [3H]Substrate transport inhibition

20 nM of [3H]vinblastine were added in each well to the BL compartment, in the absence and in the presence of P-gp inhibitors (from 200 nM to 400 μM). After 120 min at 37°C, [3H]vinblastine’s appearance was monitored at the AP compartment. At 120 min a 20 μL sample was taken from the donor compartment to determine the concentration of the residual radioligand at the end of the experiment. Samples were analyzed using LS6500 Beckman–Counter. For each compound, [3H]vinblastine transport inhibition was calculated as radioactivity difference between radioligand in the presence and absence of the given compound. These differences were expressed as inhibition rate at each drug concentration. Finally, the half maximal effective concentration (EC50) values were determined [25].

### Cell ATP availability assay

Caco-2 cells were seeded into 96-well microplate in 100 μL of complete medium at a density 2×104 cells/well. The plate was incubated O/N in a humidified atmosphere (5% CO2 at 37°C). The medium was removed and 100 μL of complete medium were added, in the presence or absence of different concentrations of test compounds. The plate was again incubated for 2 h in a humidified atmosphere. Then, 50 μl of mammalian cell lysis solution were added to all wells and the plate stirred for 5 min in an orbital shaker. In all wells, 50 μl of substrate solution were added, and the plate stirred for 5 min as above reported. The plate was dark adapted for 10 min and the luminescence measured in Victor3 (PerkinElmer) [26].

### Calcein-AM experiment

The ability of celecoxib to interact with each transporter and restore calcein-acetoxymethylester (calcein-AM) was studied in a cell system represented by MDCK cells monolayers, specifically prepared to overexpress P-gp, MRP1 or BCRP (MDCK-P-gp, MDCK-MRP1, and MDCK-BCRP). The method was adapted from Eneroth et al. [27] and Korjamo et al. [28] with minor modifications. Briefly, each cell line was seeded into a black Cultureplate (PerkinElmer) 96/wells plate with 100 μl medium and allowed to become confluent overnight. Test compounds were solubilized in 100 μl of culture medium and added. After incubation at 37°C for 30 min. Calcein-AM, a fluorescent dye, was added in 100 μl of PBS to yield a final concentration of 2.5 μM. After 30 min, each well was washed with PBS and the plate was read using Victor3 at excitation and emission wavelengths of 485 nm and 535 nm, respectively. After definition of the fluorescence basal level in untreated cells, calcein-AM cell accumulation, was measured in the presence of the tested compound (celecoxib). In treated wells the increase of fluorescence as compared to basal level was measured. The half maximal effective concentration (EC50) values were determined by fitting the fluorescence increase percentage versus log[dose] [29].

### Intracellular mitomycin C accumulation

The time course of MMC intracellular accumulation and its modulation by celecoxib were evaluated by flow cytometry. For the purpose of the experiment, MMC and celecoxib were added to UMUC-3 and UMUC-3-CX cells at EC50 concentrations of the corresponding cell lines (Table 1). After incubation, the cell medium was removed and trypsin/EDTA was used to detach the cells from the plates. Cells were harvested, washed twice in ice-cold PBS (pH 7.4), and placed on ice (<1 hour) until analysis. Analysis was performed using a 530/30 filter (FL1-H [height of fluorescence intensity]). Fluorescence measurements of individual cells were done with a Becton Dickinson FACScan equipped with an UV argon laser. Analysis was gated to include single cells, based on forward and side light scatter, and was based on the acquisition of data from 10,000 cells [30].

**Table 1:** Antiproliferative effect of CLX and MMC, alone and in co-administration at 48 h, in bladder cancer cell lines

	**UMUC3**	**UMUC-3- CX**	**5637**	**5637 si-CX**	**TCC sup**	**J82**
	**EC**_**50 **_**(μM)**					
**CLX**	14.2 ± 0.7	16.3 ± 0.8	> 50	> 50	15.7 ± 0.50	11.6 ± 0.31
**MMC**	1.69 ± 0.09	13.0 ± 1.0	0.93 ± 0.04	0.62 ± 0.05	0.83 ± 0.07	0.64 ± 0.09
**CLX 1 μM + MMC**	1.54 ± 0.1	6 ± 0.3	1.46 ± 0.10	0.59 ± 0.03	0.74 ± 0.08	0.48 ± 0.03
**CLX 5 μM + MMC**	1.75± 0.07	5.7 ± 0.2	1.31± 0.06	0.47 ± 0.01	0.68 ± 0.04	1.04 ± 0.10
**CLX 10 μM + MMC**	1.9 ± 0.1	4 ± 0.18	1.68 ± 0.11	1.01 ± 0.20	1.08 ± 0.20	1.09 ± 0.10

**EC**_**50**_ = the concentration of a compound where 50% of its maximal effect is observed.

The EC_50_ values were obtained from non-linear iterative curve fitting by Prism v.3.0, GraphPad software.

Compounds were tested at escalating doses, starting from 0.1 μM to 50 μM. Results are expressed as EC_50._

### Statistical methodology

All the reported values are expressed as means ± standard deviation (SD) from triplicate experiments. EC50 values were obtained from non-linear interative curve fitting by GraphPad, Prism. For variance estimation, *t*-test and Wilcoxon test were used. Statistical differences in Table 1 and in Table 2 were determined by Mann–Whitney unpaired test. Differences were considered statistically significant when P values were <0.05. All the research carried out and reported in the present manuscript was reviewed and approved by the ethical committee of the Ospedale Policlinico Consorziale of Bari, Italy.

**Table 2:** Biological evaluation of MDR inhibitors and reference compounds

**EC**_**50 **_**± SEM,**^***a ***^**(μM)**				
**Compounds**	**Caco-2 [**^**3**^**H] vinblastine transport inhibition**	**MDCK-MDR1 Calcein-AM**	**MDCK-MRP1 Calcein-AM**	**MDCK-BCRP Calcein-AM**
**Celecoxib**	30 ± 2.0	46.9 ± 2.5	10.1 ± 0.5	24.1 ± 1.2
**Mitomycin C**	10 ± 0.5	N.D.^*b*^	N.D.^*b*^	N.D.^*b*^
**MK-571**^***c***^			2.85 ± 0.25	
**Verapamil**^***c***^	20^*d*^			3.65 ± 0.2

^*a*^Data ± SEM are the mean of three independent determinations (samples in triplicate).

^*b*^Mitomycin C activity was not determined because in the experimental conditions quenched the spectroscopic properties of the probe.

^*c*^Reference compounds for MDR pumps.

^*d*^See Colabufo et al. [40].

## Results

### Expression of COX-2 and ABC trasporters in bladder cancer cells

**Figure 1:**
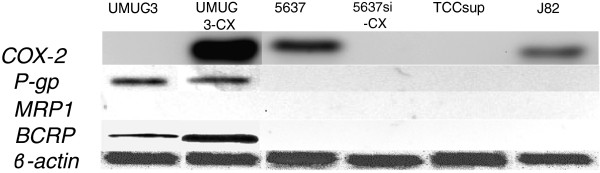
**Protein expression levels of COX-2, P-gp, MRP1 and BCRP in UMUC3, UMUC-3-CX, 5637, 5637si-CX, TCCsup, and J82, as determined by western blot.** Cell lysates were obtained from exponentially growing cells and subjected to immunoblotting with appropriate antibodies. Immunoblotting with an antibody to β-actin was used to ensure equal loading of proteins in each lane (bottom).

**Figure 2:**
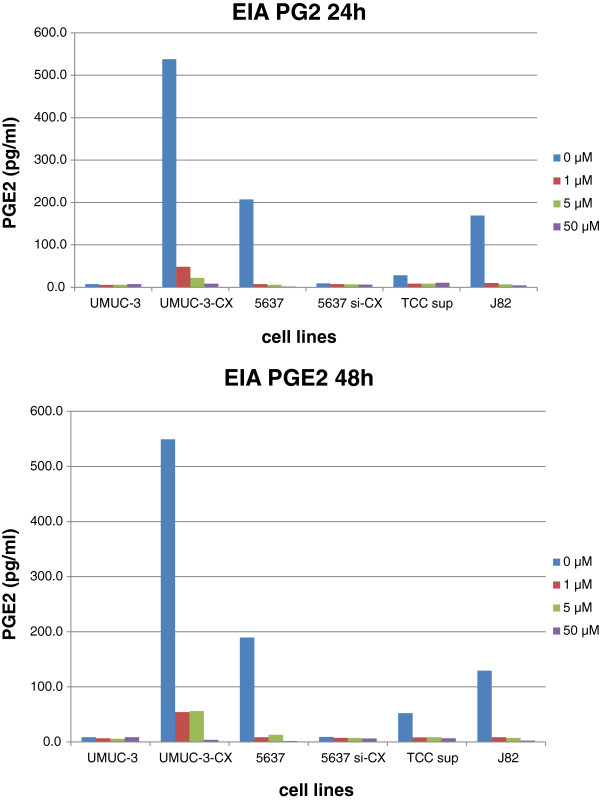
**Secretion of prostagladin E2 in the culture medium of a selection of bladder cancer cell lines.** PGE2 in cell supernatant was determined by enzyme immunoassay after treatment with or without known concentrations (0 - 1- 5–50 μM) of Celecoxib for 24 and 48 h.

COX-2 and ABC transporters expression was studied via western blot in several bladder cancer cell lines (Figure 1). As previously reported [15], UMUC-3 lacks COX-2 expression and was used to create UMUC-3-CX, a COX-2 overexpressing transfectant. Moreover, 5637 showed the highest COX-2 levels among the tested bladder cancer cells and was used to obtain a silenced non-expressing 5637si-CX cell line. Successful UMUC-3 transfection and 5637 silencing are shown in Figure 1. Among the ABC transporters, P-gp expression was similar in wild type and in transfected UMUC-3, but undetectable in the other cell lines. MRP1 protein was neglectable in all cell lines. Finally, BCRP levels were increased in UMUC-3-CX as compared to UMUC-3, while remained undetectable in all other cell lines. Additionally, COX-2 functional activity was tested in all cells by measuring PGE2 production. As expected, celecoxib administration caused a dose dependent reduction of endogenous PGE2 secretion in all, except COX-2 non-expressing cells (UMUC-3 and 5637si-CX) (Figure 2). Short-term (24 and 48 h) exposure of our cell lines to celecoxib (0.1 μM to 50 μM) caused no significant change in transporter’s expression as studied by western blot (data not shown).

### Mitomycin C anti-proliferative effect and intracellular accumulation in UMUC-3 and UMUC-3-CX cells

**Figure 3:**
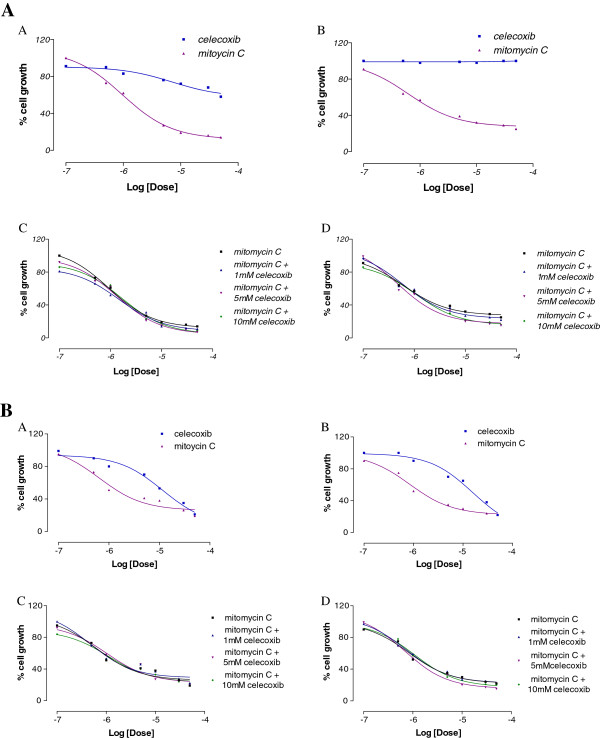
**Antiproliferative effect of celecoxib and MMC administered alone at increasing doses (0,1 - 0,5 - 1–5 - 10–30 - 50 μM) (A, B).** Antiproliferative effect of MMC alone and in co-administration with known concentrations of celecoxib (**C**, **D**). All experiments conducted after 48 h of incubation in 5637 (**A**, **C**) and in 5637si-CX (**B**, **D**) cells.

**Figure 4:**
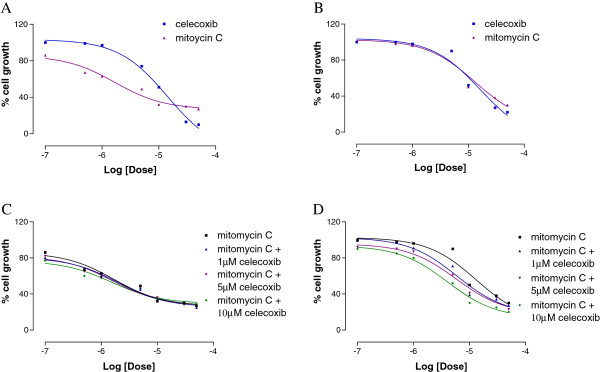
**Antiproliferative effect of celecoxib and MMC administered alone at increasing doses (0,1 - 0,5 - 1–5 - 10–30 - 50 μM) (A, B).** Antiproliferative effect of MMC alone and in co-administration with known concentrations of celecoxib (**C**, **D**). All experiments conducted after 48 h of incubation in UMUC-3 (**A**, **C**) and in UMUC-3-CX (**B**, **D**) cells.

The anti-proliferative effect of celecoxib and MMC were investigated alone and in co-administration at 48 h, in 5637 and 5637si-CX cells (Figure 3), and in UMUC-3 and UMUC-3-CX cells (Figure 4). Compounds were tested at escalating doses, starting from 0.1 μM to 50 μM. Results are expressed as EC50 and are shown in Table 1. Dose response curves are shown in the corresponding figures. When administered alone, MMC had a comparable anti-proliferative effect both in 5637 and 5637si-CX cells; in these cells celecoxib was unable to affect MMC killing (Figure 3). However, in the same experimental conditions, the effect of MMC was eight fold greater in UMUC-3 as compared to UMUC-3-CX (Figure 4). Importantly, the co-administration of 1 μM, 5 μM, and 10 μM celecoxib to MMC did not cause a significant increase of MMC cytotoxicity in UMUC-3 cells (Figure 4C). By contrast, in UMUC-3-CX cells, the anti-proliferative activity of MMC was 2–3 fold improved by the co-administration of celecoxib compared to MMC alone (Table 1, and Figure 4D).

**Figure 5:**
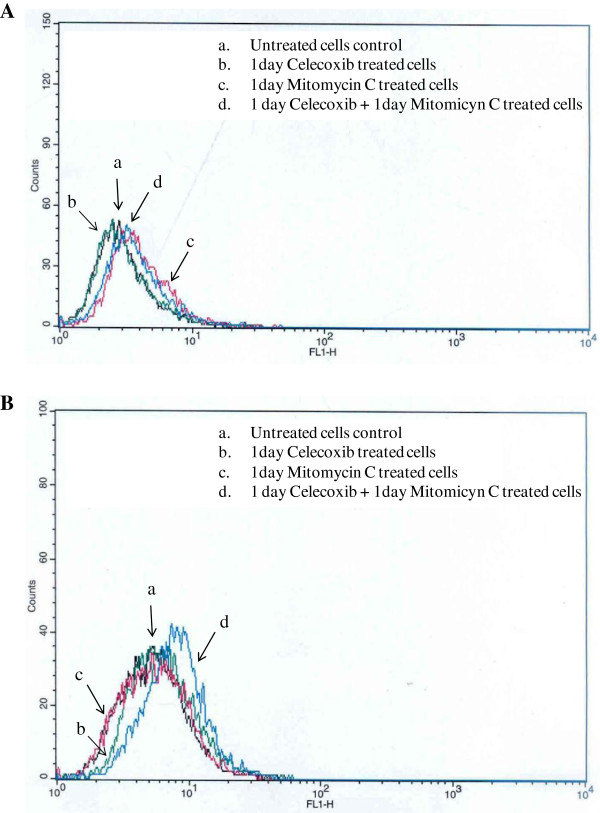
**Flow cytometry analysis to study the time course of MMC intracellular accumulation and its modulation by celecoxib in UMUC-3 (A) and UMUC-3-CX (B).** In both cases, cells were treated one day with MMC (1.69 μM) or celecoxib (14.2 μM) alone, or with both in co-administration (MMC 1.69 μM + celecoxib 14.2 μM). Celecoxib causes an increase in the intracellular concentration of MMC in UMUC-3-CX, but not in UMUC-3 cells.

Since UMUC-3 transfection caused an overexpression of BCRP in UMUC-3-CX cells, we sought to explore the ability of celecoxib to interfere with the efflux of MMC. For this purpose, flow cytometry was performed to study MMC intracellular accumulation, both in UMUC-3 and UMUC-3-CX cells. The cells were either treated one day with MMC or celecoxib alone, or with both in co-administration using the following sequential schedule: 1 day celecoxib, and afterwords, 1 day MMC and celecoxib added to the same plate (Figure 5). Importantly, celecoxib was able to produce an increase of intracellular MMC concentration only in UMUC-3-CX cells, as shown by the right shift of the d curve as compared to the c curve (Figure 5B). This effect was not seen in UMUC-3 cells (Figure 5A).

### Celecoxib-transporter interacting mechanism

Three biological assays (Drug Transport Experiment, Substrate Transporter Inhibition, and ATP cell depletion) were combined to establish if there was an interaction between P-gp and celecoxib. In the drug transport experiment, a Caco-2 cell monolayer was used to determine the apparent permeability (Papp) of celecoxib, both as basolateral-apical flux (B→A) and apical-basolateral flux (A→B). Celecoxib displayed Papp A→B = 137 nm/sec, Papp B→A = 1027 nm/sec, and a 7.5 BA/AB ratio. This result suggests that celecoxib is effluxed by P-gp. Indeed, compounds displaying BA/AB ratio > 2 are linked and flipped to the extra cellular compartment by pumps, while drugs with BA/AB ratio < 2 are not transported. Moreover, the Subtrate Transporter Inhibition experiment was performed to study the modulating effect of celecoxib on [3H]vinblastine transport. Compared to Verapamil, which is the reference compound for this experiment, celecoxib had a similar EC50 value (EC50 = 30 ± 2.0 μM) (Table 2). Finally, when tested in Caco-2 cells to monitor ATP cell depletion, celecoxib decreased ATP levels in a time- and dose-dependent fashion. As a result of all finding combined, celecoxib should be classified as P-gp unambiguous substrate [24].

### Celecoxib in MDCK cells overexpressing P-gp or BCRP or MRP1 pumps

**Figure 6:**
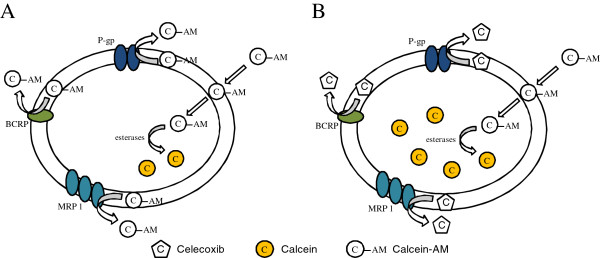
Schematic view of the mechanism of calcein cellular retention in the absence (A) and in the presence (B) of an MDR pump substrate, such as celecoxib.

In MDCK-P-gp, in MDCK-MRP1 and in MDCK-BCRP cells the ability of celecoxib to restore calcein-AM cell accumulation was tested. This dye, calcein-acetoxymethylester, is a non fluorescent lipophilic P-gp/MRP1/BCRP substrate that diffuses across the plasma membrane into the cell where is hydrolyzed into highly fluorescent calcein by endogenous cytoplasmic esterases. This fluorescent compound is not effluxed by P-gp, MRP1, BCRP and it cannot cross the cell membrane via passive diffusion because of its hydrophilie. Therefore, in the presence of an MDR transporter modulator, calcein retention and a rapid fluorescence increase is monitored (Figure 6). In Table 2, calcein cell accumulation is plotted vs Log[celecoxib] and the following results are displayed: in MDCK-P-gp cells EC50 = 46.9 ± 2.5 μM, in MDCK-MRP1 EC50 = 10.1 ± 0.5 μM, and in MDCK-BCRP EC50 = 24.1 ± 1.2 μM. Compared to the reference compounds, these findings confirm that celecoxib is a substrate for P-gp, MRP1 and BCRP pumps.

## Discussion

In recent years, there has been an interest to investigate the potential link between COX-2 expression and the development of MDR in multiple tumors, including urothelial cancer [21,22]. A direct causal link between the activation of the COX-2/PGE2 signal pathway and the up-regulation of all three ABC transporters has been documented [31-35] and supports the strong correlation between COX-2 and MDR1/P-gp expression seen in several tumor specimens [36-38]. Overexpression of P-gp has been found in human bladder cancer cells selected by drug resistance against P-gp-targeting drugs [39-41]. In patients with bladder cancers, expression of P-gp is often increased after chemotherapeutic treatment [42]. Prophylactic intravesical instillation of MMC, doxorubicin and epirubicin has been useful for reducing recurrence of NMIBC; however, recurrence rates are still high and progression to more invasive disease is not affected even after intensive intravesical chemotherapy [43].

In this study we developed an *in vitro* bladder cancer model to study if COX-2 inhibitors can modulate tumor resistance to MMC by interfering with the activity of membrane transporter proteins of the ABC family. For this purpose we used UMUC-3 cells, constitutively lacking COX-2 expression, and UMUC-3-CX cells, in which COX-2 is overexpressed. When MMC was administered alone, UMUC-3-CX cells resulted resistant to MMC killing. However, for the first time we showed that pre-treatment with a selective COX-2 inhibitor, celecoxib, caused a significant and dose dependent increase in the cytotoxic activity of MMC. Interestingly, in UMUC-3 cells MMC activity was not affected by celecoxib. Moreover, compared to UMUC-3, we found that forced COX-2 overexpression in UMUC-3-CX cells increased PGE2 production and up-regulated BCRP, one of the transporters involved in MDR. These data were confirmed by the observation of an increase in intracellular concentration of MMC when UMUC-3-CX cells were co-treated with celecoxib. Again, intracellular MMC concentration was not affected by celecoxib in UMUC-3 cells. Although several causes may be taken into account it has been shown that ABC transporters, such as BCRP, induce drug resistance by promoting drug efflux out of the cells [44]. Indeed, when the cytotoxicity properties of MMC were studied in a cell line completely lacking any ABC transporter expression, such as 5637 and 5637si-CX cells, celecoxib administration was unable to affect MMC killing. Assuming a causal link between COX-2 expression and MDR, COX-2 inhibitors would be expected to prevent ABC transporters induction and sensitize cells to antineoplastic agents. This has been previously shown in Caco-2 cells where indomethacin, nimesulide and naproxen directly reduced MRP1 expression and P-gp relative amount and function [45]. Similarly, in human lung cancer cells celecoxib was shown to downregulate the expression of MRP1 [19].

Although COX-2 enzyme inhibition could not be excluded in our experimental model, we sought to investigate if the effect seen in UMUC-3-CX cells after celecoxib administration could be the result of a direct interaction between celecoxib and any of the three transporters involved in MDR. To explore this hypothesis specific biological assays were performed to demonstrate that celecoxib is a substrate for the MDR transporters explored in this study. Our data demonstrate that celecoxib is effluxed by P-gp, BCRP and MRP1 pumps and causes a time- and dose-dependent ATP cell depletion in Caco-2 cells. Further, celecoxib competes with and may inhibit the transport of other reference drugs (vinblastine in our experimental model) (Table 2). Finally, the ability of celecoxib to restore Calcein-AM cell accumulation in MDCK-P-gp, MDCK-MRP1 and MDCK-BCRP cells suggests that celecoxib is a substrate for all the transporters tested in our study. Thus, the increase in MMC concentration seen in UMUC-3-CX after co-administration with celecoxib may be the result of a transporter-celecoxib interaction. As a hypothesis, since BCRP was the only transporter overexpressed by UMUC-3-CX cells, a BCRP-celecoxib interaction may justify the greater anti-proliferative activity obtained when MMC and celecoxib were co-administered (Figures 3 and 4).

Our findings are in agreement with previous studies in which COX-2 inhibitors have shown to produce MDR-regulating effects that are COX-2 independent. Van Wijngaarden and co-workers proposed that the effects of celecoxib were most likely mediated by inhibition of NF-kb and not related to COX-2 or the activity of the ABC transporters P-gp, MRP1 and ABCG2 [46]. Likewise, Ye et al. found that indomethacin and SC236, a COX-2-selective inhibitor, sensitized human hepatocellular carcinoma HepG2 cells to the cytotoxicity of doxorubicin by significantly increasing doxorubicin intracellular accumulation; however, the effects were not reversed by prostaglandin E2, implicating a COX-independent mechanism [47]. Taken together, these findings suggest that COX-2-selective inhibitors may enhance the effect of certain anti-cancer agents and overcome drug resistance through mechanisms that by-pass the COX-2 cascade.

Many reports examining the clinical benefits of COX-2 inhibitors and nonsteroidal anti-inflammatory drugs (NSAIDs) have addressed the role of these compounds in bladder chemoprevention. *In vitro* and in vivo research suggests that NSAIDs hinder growth and survival of bladder cancer cells [48,49], however, epidemiologic studies investigating the association between non selective NSAIDs and bladder cancer have been conflicting, with two large cohort studies suggesting that non-aspirin NSAIDs, but not aspirin, may protect from bladder cancer [50,51]. Such conflicting data have discouraged the scientific community to promote prospective clinical testing of aspirin or acetic acids (indomethacin, sulindac) in patients with NMIBC. On the other hand, the body of evidence pointing to the role of COX-2 pathway in the development of bladder cancer is compelling and supported by several epidemiologic studies [51]. More recently, COX-2 selective inhibitors have been the focus of much scrutiny in terms of cardiovascular risk. Of such inhibitors, celecoxib is thought to have a better safety profile, and has been tested in patients with NMIBC, and clinical trials are under way. Sabichi et al. recently reported the results of a phase IIb randomized controlled trial of celecoxib to prevent recurrence following TUR in 146 patients with NMIBC [52]. Although the primary endpoint (time to recurrence) did not reach statistical significance, the results support a beneficial effect of celecoxib in such patients. For the ultimate interpretation of these results, the ongoing phase III randomized controlled Bladder COX Inhibition Trial (BOXIT) of the same daily dose of celecoxib versus placebo is being conducted [http://www.cancerresearchuk.org].

We recognize this is a pilot study, in which we show that celecoxib is a substrate for ABC transporters and may enhance the activity of cytotoxic drugs such as MMC. However, translational studies and, more importantly, ongoing and future clinical trials will have to confirm the preclinical potential of celecoxib and other COX-2 inhibitors in NMIBC.

## Conclusions

Compared to the *wt* counterpart, the UMUC-3-CX cell line resembles a more aggressive phenotype with a higher BCRP protein expression and a low response to MMC. Interestingly, the cytotoxic activity is regained when MMC is administered in combination with celecoxib. Although COX-2 enzyme inhibition cannot be excluded, this result may partly depend on a direct interaction between celecoxib and any of the transporters involved in MDR. Indeed, we have shown that celecoxib is a substrate for P-gp/BCRP/MRP1 transporters and may modulate their activity. These findings and others from recent reports imply that the therapeutic approach of combining conventional chemotherapy with selective COX-2 inhibitors seems promising and warrants prospective clinical evaluation in patients with NMIBC in which COX-2 is overexpressed.

## Competing interests

The authors declare that they have no competing interests.

## Authors’ contribution

VP substantially contributed the study conception and design, to the analysis and interpretation of the data, to the manuscript drafting. PA carried out all cell line studies, including western blot, PGE2 assays and MTT tests. MN carried out all permeability experiments and contributed to data analysis and interpretation. NAC participated in the design of the study and performed the statistical analysis. MC and AA performed all flow cytometry studies. LC and AP participated in coordinating the study and helped to draft the manuscript. All authors read and approved the final manuscript.
